# Right Coronary Artery Dissection After Ross Procedure

**DOI:** 10.1016/j.atssr.2022.10.016

**Published:** 2022-10-29

**Authors:** Devon Anderson, Jeffrey Southard, Bob Kiaii, Gary W. Raff

**Affiliations:** 1 Division of Cardiothoracic Surgery, University of California Davis Medical Center, Sacramento, California; 2 Division of Cardiology, University of California Davis Medical Center, Sacramento, California

## Abstract

The Ross procedure is a surgical option for the treatment of aortic valve stenosis that is performed in a select subset of patients. This case report highlights the rare complication of a coronary artery dissection that occurred in the early postoperative period after a Ross procedure. The importance of timely recognition, swift intervention, and multidisciplinary team collaboration is discussed in the postoperative management of this complex cardiac surgery patient.

Patients who present for surgical intervention of aortic valve stenosis receive a mechanical valve, bioprosthetic valve, aortic valve homograft, or pulmonary autograft (also known as the Ross procedure).^1^ There are risks and benefits associated with each procedure and valve type. Postoperative complications of aortic valve surgery include hemorrhage, sepsis, heart block, arrhythmias, stroke, and reexploration for bleeding.^2^ A less common postoperative complication is coronary artery dissection. We describe the rare complication of a right coronary artery (RCA) dissection that manifested in the early postoperative period after the patient underwent a Ross procedure.

A 47-year-old woman with a history of hyperlipidemia, prediabetes, and known congenital bicuspid aortic valve was found to have severe symptomatic aortic valve stenosis. Outpatient echocardiography revealed a mean gradient of 46 mm Hg across the valve, aortic valve area of 0.6 cm^2^, and ejection fraction of 66%. Symptoms included shortness of breath, chest pain with exertion, and presyncope. Preoperative cardiac catheterization confirmed severe aortic stenosis and normal left ventricular and right ventricular function with mild (30%) stenosis of the proximal RCA.

After multidisciplinary conferences, she underwent an uncomplicated supported Ross procedure. The pulmonary autograft was harvested and sewn into a 26-mm Dacron graft to replace the aortic root, and she received a 23-mm pulmonary homograft to restore continuity between the right ventricle and branch pulmonary arteries. The left main coronary artery was noted to come off close to the commissure in the bicuspid aortic root; therefore, it was anastomosed close to the commissure of the autograft after a circular opening was made in the Dacron graft and pulmonary autograft wall. The RCA was anastomosed in a similar fashion. Coronary ostial cannulation was performed during the implantation of the coronary buttons for the delivery of cardioplegia. After the procedure was completed, transesophageal echocardiography showed good biventricular function without aortic insufficiency or pulmonary insufficiency. Total cardiopulmonary bypass time and cross-clamp time were 196 and 126 minutes, respectively. Postoperatively, she was taken to the cardiothoracic intensive care unit for recovery and was extubated without complications.

On postoperative day (POD) 1, she was hypotensive with increased central venous pressures and increasing pressor requirements. Bedside echocardiography showed poor right ventricular function compared with intraoperative echocardiography. She was urgently taken to the cardiac catheterization laboratory and found to have 70% proximal RCA stenosis due to RCA dissection as seen on intravascular ultrasound (Figure 1). She underwent percutaneous coronary intervention to the proximal/ostial RCA with a drug-eluting stent, with excellent angiographic results (Figure 2). Shortly thereafter, she demonstrated tamponade physiology and was found to have a large pericardial effusion on bedside ultrasound. She was emergently transported to the operating room for mediastinal exploration. She was found to have an extremely tense and dilated right atrium with bleeding from the inferior vena cava cannulation site, which was repaired with a suture. After the operation, she was placed on venoarterial extracorporeal membrane oxygenation and transferred to the cardiothoracic intensive care unit for recovery. Four days after the index procedure, she was successfully weaned from extracorporeal membrane oxygenation. She was extubated on POD 8 and discharged on POD 15.

**Figure 1:**
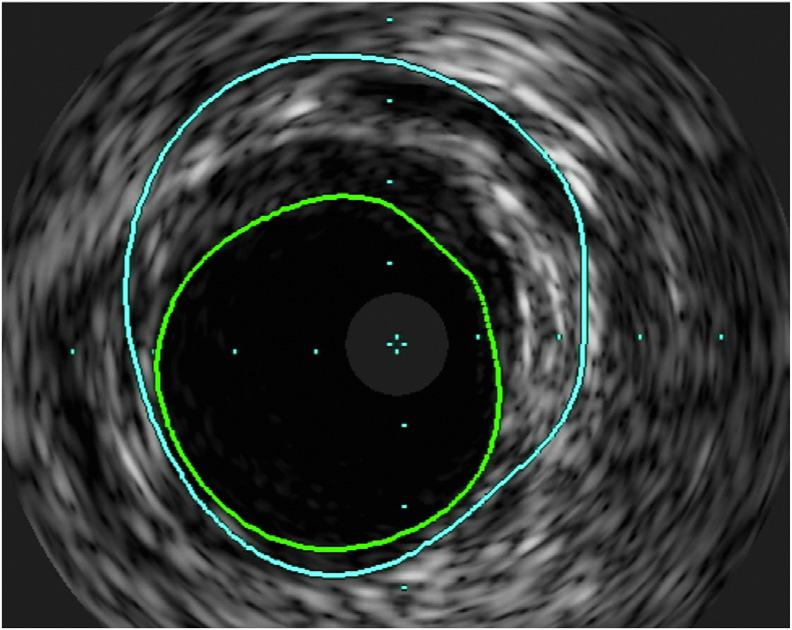
Intravascular ultrasound image showing right coronary artery dissection (green, area of coronary artery lumen; blue, area of dissection plane).

**Figure 2:**
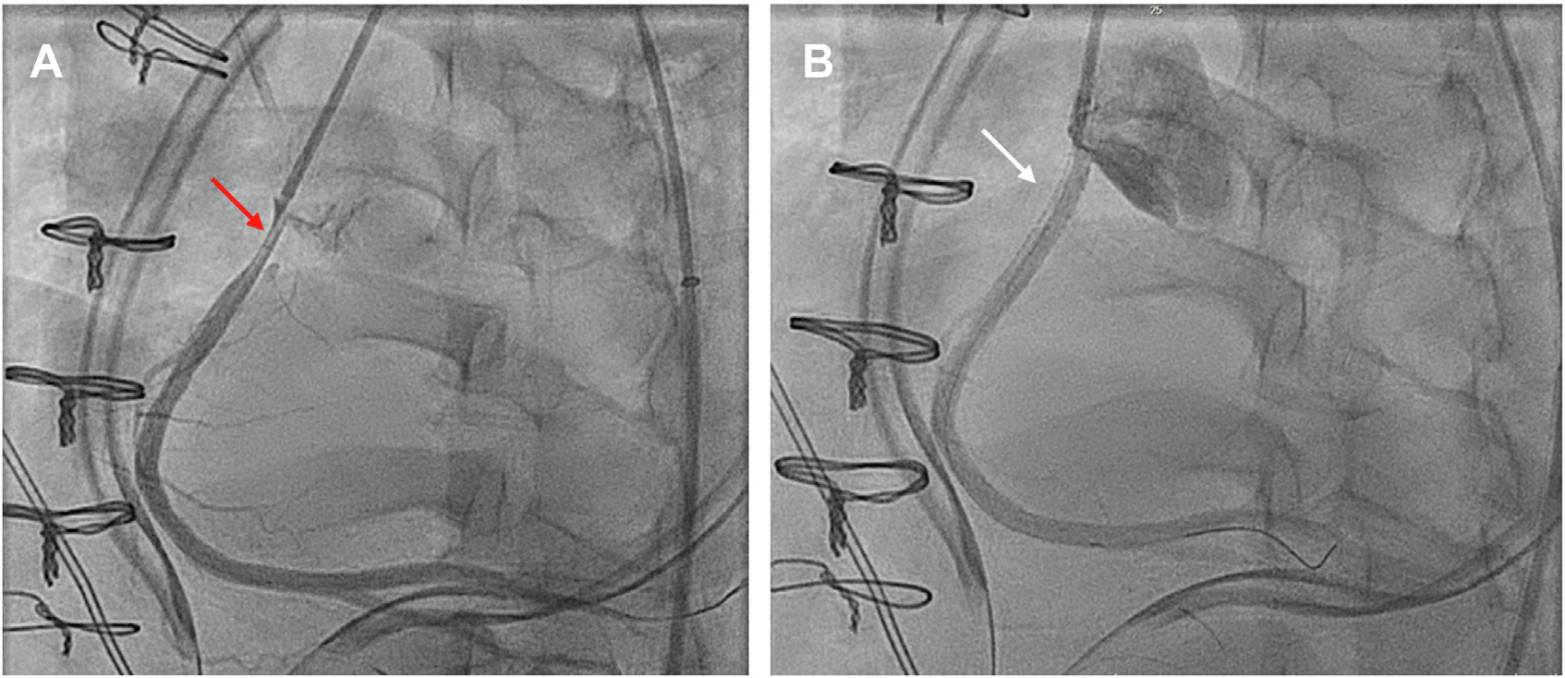
Cardiac catheterization (A) before and (B) after percutaneous intervention with drug-eluting stent (red arrow showing 70% proximal right coronary artery stenosis; white arrow after stent placement).

## Comment

The Ross procedure, first described by Donald Ross in 1967, involves replacing the aortic valve with a pulmonary autograft and placing a homograft in the pulmonary position.^1^^,^^3^ The advantages of replacing the aortic valve with a pulmonary autograft include low valve gradients and the freedom from anticoagulation.^4^^,^^5^ Studies have shown an improved life expectancy and better quality of life with the Ross procedure compared with other forms of aortic valve replacement.^4^^,^^5^ This procedure is ideal for young (≤50 years) patients with isolated aortic valve disease, small annulus, and active lifestyle and for those who wish to avoid anticoagulation and to have long-term freedom from redo operation.^1^ Despite the benefits associated with the Ross procedure, The Society of Thoracic Surgeons National Database showed that <0.5% of the aortic valve procedures performed between 1994 and 2010 were Ross procedures.^5^ This is in part due to the technical complexity of the Ross procedure as well as the wide range of morbidity and mortality associated with the operation. Single centers of excellence have reported <1% mortality, whereas other meta-analysis papers have reported 3.2% mortality.^5^ Other known deterrents to this procedure include the risk of reintervention and giving the patient “2-valve disease” with late autograft valve failure and the need for replacement of the pulmonary homograft.^1^^,^^2^^,^^6^ Dilation of the neoaortic root with subsequent autograft insufficiency can lead to a redo aortic root surgery or Bentall procedure, which is a high-risk operation in patients who have previously undergone cardiac surgery. This can be mitigated by performing the procedure in patients with a small annulus and externally reinforcing the pulmonary autograft with a Dacron graft, which is called a supported Ross procedure.^4^^,^^6^ The wide range of mortality, the risk of reintervention, and the complexity associated with this operation account for the low number of Ross procedures performed by surgeons.

Dissection of the RCA can be iatrogenic after manipulation around the coronary artery ostium or occur spontaneously. In spontaneous coronary artery dissection, an intramural hematoma creates a false lumen in the arterial wall and compresses the true lumen, which obstructs coronary blood flow.^7^^,^^8^ An intimal tear is not always visible in patients with spontaneous coronary artery dissection as it may be due to ruptured vasa vasorum bleeding into the arterial wall.^7^ The decreased blood flow can lead to myocardial infarction and is seen in younger women and usually not associated with atherosclerotic plaques.^7^^,^^8^ Iatrogenic coronary artery dissection occurs during instrumentation, such as during a cardiac catheterization or with the antegrade cardioplegia catheter during cardiac surgery. The intima of the vessel is disrupted, which leads to the dissection of tissues that can propagate into the ascending aorta, into the arch, or around the pericardium, causing cardiac tamponade.

Our patient had an RCA dissection and RCA flow disruption on POD 1. This resulted in right ventricular failure and elevated central venous pressure, which led to dilation of the right atrium and disruption of the inferior vena cava cannulation suture site, leading to cardiac tamponade. This unusual sequence of events probably resulted after instrumentation of the coronary ostium during delivery of cardioplegia in a diseased vessel. The timing suggests that there was progression of the dissection or late dissection of the vessel in the early postoperative period.

Certain postoperative complications associated with aortic valve surgery are well described. However, this case report highlights the rare complication of an RCA dissection that occurred in the early postoperative period after a supported Ross procedure. The importance of multidisciplinary team collaboration, timely recognition, and expeditious intervention highlights valuable aspects of the postoperative management of complex cardiac surgery patients. These operations should be performed at centers of excellence to mitigate and to overcome such complications.

## References

[1] Ouzounian M., Mazine A., David T.E. (2017). The Ross procedure is the best operation to treat aortic stenosis in young and middle-aged adults. J Thorac Cardiovasc Surg.

[2] Karamlou T., Jang K., Williams W.G. (2005). Outcomes and associated risk factors for aortic valve replacement in 160 children: a competing-risks analysis. Circulation.

[3] Ross D.N. (1967). Replacement of aortic and mitral valves with a pulmonary autograft. Lancet.

[4] Buratto E., Shi W.Y., Wynne R. (2018). Improved survival after the Ross procedure compared with mechanical aortic valve replacement. J Am Coll Cardiol.

[5] Tam D.Y., Wijeysundera H.C., Ouzounian M., Fremes S.E. (2019). The Ross procedure versus mechanical aortic valve replacement in young patients: a decision analysis. Eur J Cardiothorac Surg.

[6] David T.E., David C., Woo A., Manlhiot C. (2014). The Ross procedure: outcomes at 20 years. J Thorac Cardiovasc Surg.

[7] Main A., Saw J. (2019). Percutaneous coronary intervention for the treatment of spontaneous coronary artery dissection. Interv Cardiol Clin.

[8] Tweet M.S., Akhtar N.J., Hayes S.N., Best P.J., Gulati R., Araoz P.A. (2019). Spontaneous coronary artery dissection: acute findings on coronary computed tomography angiography. Eur Heart J Acute Cardiovasc Care.

